# Diagnostic Challenges and Management of Crohn’s Disease: The Value of Capsule Endoscopy

**DOI:** 10.7759/cureus.106214

**Published:** 2026-03-31

**Authors:** Samone Alexander, Iyawnna Hazzard, Arleign-Ann Byer, Yinka Davies

**Affiliations:** 1 Department of Research, California Northstate University College of Medicine, Elk Grove, USA; 2 Department of Pediatric Gastroenterology, Sacramento Pediatric Gastroenterology, Sacramento, USA; 3 Department of Medicine, California Northstate University College of Medicine, Elk Grove, USA

**Keywords:** capsule endoscopy, colonoscopy, crohn’s disease, esophagogastroduodenoscopy, inflammatory bowel disease

## Abstract

Crohn’s disease (CD) can be difficult to diagnose, particularly in its early stages when symptoms lack chronicity. This report describes a male patient with persistent right lower quadrant (RLQ) pain and weight loss despite an appendectomy. Despite normal biopsies and imaging, symptoms continued, prompting further evaluation with capsule endoscopy (CE), which revealed distal small bowel inflammation supporting the diagnosis of CD. Treatment with mesalamine and a specific carbohydrate diet (SCD) led to symptom resolution. However, after discontinuing treatment, the patient developed disease progression and complications. This case underscores the limitations of standard diagnostics and the value of CE in detecting early small bowel CD and highlights the importance of sustained maintenance therapy to prevent progression.

## Introduction

Crohn’s disease (CD) is a chronic, inflammatory bowel disease (IBD) affecting the small and large intestines in a skip-segmental and transmural pattern [1]. It is more prevalent in Western countries and has a rising global incidence and is linked to risk factors such as tobacco use and childhood antibiotic exposure [1]. Common symptoms include right lower quadrant (RLQ) pain, chronic watery diarrhea, fatigue, weight loss, and perianal abscesses [2], while extraintestinal manifestations may involve arthritis, dermatological findings, ocular inflammation, and oral ulcers [3].

Diagnosis is typically based on a combination of clinical findings, imaging, and histopathology obtained through esophagogastroduodenoscopy (EGD), colonoscopy, capsule endoscopy (CE), or magnetic resonance enterography (MRE) [4]. While EGD and colonoscopy allow for biopsies and histological analysis, these are limited to specific areas of the intestine and do not survey the entirety of the small and large bowel. Additionally, they only biopsy the superficial mucosal layers and cannot assess the full intestinal wall [5]. EGD evaluates the oropharynx to the proximal part of the duodenum [6], whereas colonoscopy is able to visualize the internal anus up to the terminal ileum [7]. A large remainder of the small intestine remains unevaluated, representing a substantial limitation of these methods and potentially overlooking the diagnosis of IBD.

MRE is useful for evaluating transmural and extraluminal disease, whereas CE provides direct visualization of the mucosa throughout the small bowel. Therefore, CE plays a critical role in cases where standard diagnostic methods are inconclusive.

CE offers a less invasive method to detect early small bowel lesions or erythema, especially in the jejunum and proximal ileum, which are often missed by standard imaging [8]. The limitation of CE is that it only visualizes the mucosa and carries a risk of retention in cases of small bowel narrowing. However, it provides high-resolution visualization of the mucosa [9]. In patients with suspected strictures or prior abdominal surgery, a patency capsule may be considered prior to CE to reduce the risk of retention. This case illustrates an atypical diagnostic path for CD, emphasizing early detection and the importance of sustained treatment to prevent complications.

## Case presentation

A 28-year-old male initially presented to the clinic at age 16 in June 2012 with persistent weight loss and RLQ abdominal pain following appendectomy. Although the appendix appeared focally erythematous, pathology revealed no intraluminal exudates or inflammation. In June 2013, EGD and colonoscopy showed normal mucosa without ulceration or nodularity. Biopsies revealed mild gastritis but were otherwise unremarkable. Despite these normal findings, symptoms persisted with continued RLQ pain. An MRE performed in early 2014 was also unremarkable.

Given persistent symptoms despite negative initial evaluations, a CE was performed, revealing gastritis and mild erythema in the distal small intestine, prompting a presumptive diagnosis of CD. Repeat EGD and colonoscopy in December 2016 demonstrated chronic inflammation in the gastric antrum, superficial small bowel villous changes, and focal active colitis. Based on the segmental nature of inflammation, a diagnosis of CD was established. He was started on mesalamine and a specific carbohydrate diet (SCD), with symptomatic improvement. Repeat evaluation one year after initiating treatment revealed normal MRE and CE. He relocated and subsequently discontinued treatment, believing he was in remission.

**Figure 1:**
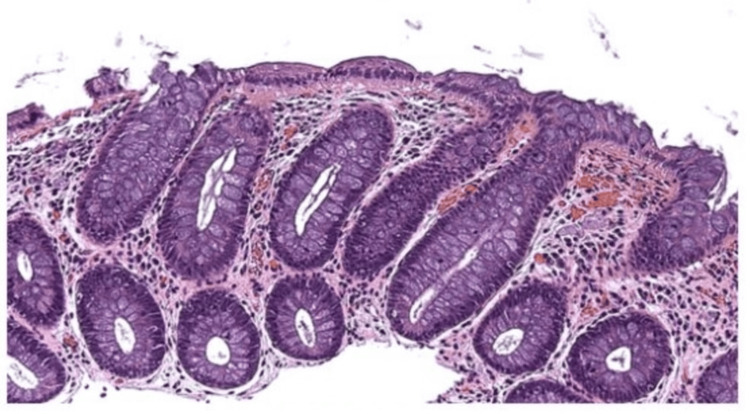
Small bowel and colon biopsy findings Evidence of benign lymphoid aggregates in the ileal and colonic mucosa, without evidence of chronic, active, lymphocytic, or collagenous colitis (March 2023).

**Figure 2:**
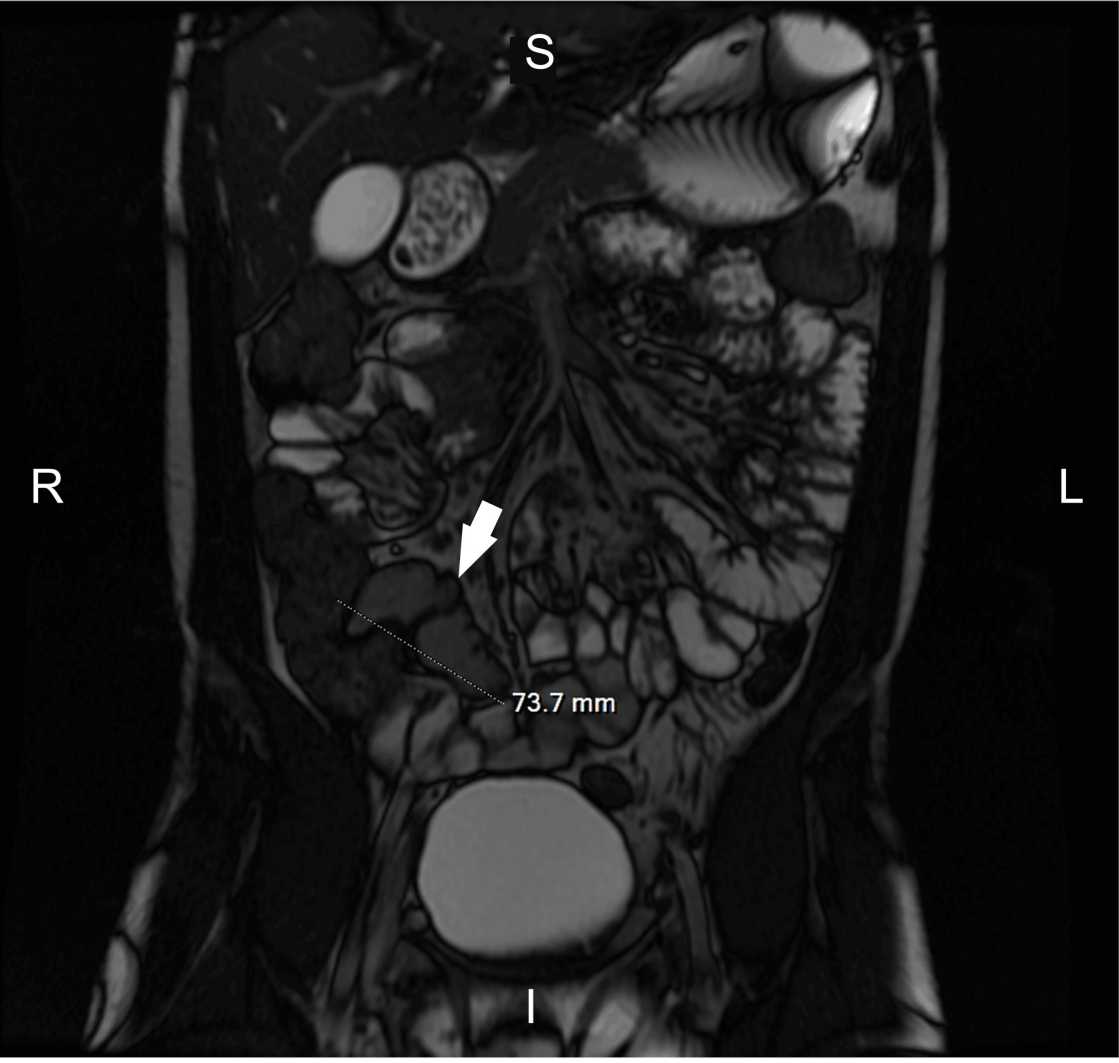
Magnetic resonance enterography (MRE) findings An 8 cm of small bowel wall thickening (arrow). Five years after diagnosis in April 2023. R: right; L: left; S: superior; I: inferior

Approximately one year later, he presented to the emergency department with severe RLQ pain, diarrhea, and hematochezia. MRE revealed a 5 cm segment of small bowel narrowing with transmural hyperenhancement and restricted diffusion. Computed tomography showed wall thickening in the descending and sigmoid colon and a fluid collection suggestive of an abscess. He was treated with intravenous methylprednisolone and antibiotics, then transitioned to oral prednisone and mesalamine. Repeat biopsy in March 2023 showed benign lymphoid aggregates without active colitis (Figure 1), and follow-up MRE demonstrated 8 cm of small bowel thickening, indicating disease progression (Figure 2).

## Discussion

This case highlights the importance and necessity of using multiple diagnostic modalities for the diagnosis and monitoring of CD; no single diagnostic tool is sufficient. The case also highlights the importance of maintenance treatment to prevent disease progression. The patient’s discontinuation of treatment, despite experiencing symptom resolution and mucosal healing, emphasizes the importance of sustained, long-term management for CD. CD’s unpredictable relapsing-remitting nature indicates that even when symptoms temporarily subside, patients can develop cumulative structural damage to the bowel over time [10]. Thus, patients may have ongoing subclinical inflammation despite apparent clinical remission. However, without continuous pharmacological management, ongoing mucosal inflammation increases the risk of life-threatening complications, as demonstrated in this case. Therefore, consistent treatment is essential to prevent these complications.

Previous studies have shown that untreated CD leads to faster progression of mucosal damage driven by macrophage-mediated inflammation [11]. This inflammation can lead to intestinal stricture formation, increasing the risk of obstructive episodes [12]. Research also shows that CD is one of the leading causes of small bowel obstruction [13]. Together, prior literature and this case reinforce the necessity of continued treatment even during asymptomatic periods to mitigate complications and maintain mucosal healing.

In patients with CD, where traditional diagnostic methods fail to identify the disease, CE can be an important tool for enabling earlier diagnosis and treatment. In this patient, the initial MRE was unremarkable, whereas CE identified distal small bowel erythema, highlighting a key difference in diagnostic capability. Unlike MRE, which primarily evaluates transmural and extraluminal disease, CE provides direct imaging of the small bowel mucosa and has a high sensitivity for detecting subtle mucosal lesions characteristic of CD [14]. Previous studies report a high diagnostic yield for CE, approximately 70%, in suspected small bowel CD, particularly when traditional tests are inconclusive [8]. Therefore, CE can help detect otherwise missed lesions and prompt earlier diagnosis and intervention.

This case illustrates the significance of CE, especially given the patient's initial presentation. Although the patient's initial treatment involved an appendectomy, research suggests that appendectomy alone may exacerbate CD progression by triggering intestinal dysbiosis, potentially leading to subsequent inflammation [15]. Additionally, persistent symptoms despite negative initial evaluations justified further investigation with CE in this case. This further reinforces the importance of comprehensive follow-up when subtle abnormalities are present. CE offers a minimally invasive, anesthesia-free diagnostic option that can reveal early or atypical disease manifestations. When used appropriately, it provides timely diagnostic insight and may improve long-term outcomes in patients with suspected or early CD.

## Conclusions

This case underscores the importance of a multimodal diagnostic approach for early diagnosis and intervention in CD. It also reinforces the role of advanced diagnostic tools such as CE in detecting early small bowel disease that may be missed by conventional imaging. As demonstrated by this case, patient education and adherence to sustained, long-term management are critical in preventing disease progression and complications.
